# Factors Influencing the Efficacy of COVID-19 Vaccines: A Quantitative Synthesis of Phase III Trials

**DOI:** 10.3390/vaccines9040341

**Published:** 2021-04-01

**Authors:** Luigino Calzetta, Beatrice Ludovica Ritondo, Angelo Coppola, Maria Gabriella Matera, Nicola Di Daniele, Paola Rogliani

**Affiliations:** 1 Respiratory Disease and Lung Function Unit, Department of Medicine and Surgery, University of Parma, 43125 Parma, Italy; 2 Unit of Respiratory Medicine, Department of Experimental Medicine, University of Rome “Tor Vergata”, 00133 Rome, Italy; beatriceritondo@gmail.com (B.L.R.); coppolangelo@gmail.com (A.C.); paola.rogliani@uniroma2.it (P.R.); 3 Department of Experimental Medicine, University of Campania Luigi Vanvitelli, 80138 Naples, Italy; mariagabriella.matera@unicampania.it; 4 Department of Systems Medicine, University of Rome “Tor Vergata”, 00133 Rome, Italy; didaniele@med.uniroma2.it

**Keywords:** COVID-19, efficacy, meta-regression, modifying factors, SARS-Cov-2, vaccine

## Abstract

To date, there is still a paucity of data from Phase III trials concerning the efficacy of vaccines against COVID-19. Furthermore, no studies investigated the variables that may modulate the efficacy of vaccination. The aim of this analysis was to assess whether there are modifying factors that may potentially influence the clinical efficacy of COVID-19 vaccines. A quantitative synthesis of data from Phase III trials was performed via pairwise and network meta-analyses, along with meta-regression analysis. Data from Phase III trials are currently available only for AZD1222, BNT162b2, mRNA-1237, and Sputnik V. Vaccination resulted to be generally effective (90.0%, 95%CI 72.6–96.4; *p* < 0.001), although the efficacy of AZD1222 (62.1%) introduced a significant level of heterogeneity in the meta-analysis (I^2^ 92.17%, *p* < 0.001). No significant modifying factors resulted from the meta-regression analysis. However, considering the mRNA-based vaccines, a trend toward significance (*p* = 0.081) resulted for age. The network meta-analysis provided the following rank of effectiveness: BNT162b2 ≃ mRNA-1273 > Sputnik V >> AZD1222. In conclusion, no modifying factors seem to modulate the efficacy of vaccines against COVID-19. This quantitative synthesis will need to be updated as soon as further clinical results on the efficacy profile are available from Phase III trials for further licensed COVID-19 vaccines.

## 1. Introduction

From late 2019, the outbreak of severe acute respiratory syndrome coronavirus 2 (SARS-CoV-2) has spread restlessly across the globe, causing 109 million confirmed cases and almost 2.5 million deaths [1]. In this scenario, testing of new vaccines against Coronavirus Disease 2019 (COVID-19) has been expedited, along with the authorization process, the production, and the deployment of vaccines, despite the logistic hurdles straining supply chains to the limit [2,3,4].

Currently, 63 candidate vaccines are at clinical-stage of development, all based on several different platforms, from classical to new-generation approaches [5]. According to the World Health Organization (WHO), 22 COVID-19 vaccines are in Phase III development [6]. However, only 18 of them are being tested in Phase III trials, as four candidates are currently tested in Phase II segment of a combined Phase II/III trial [7,8,9,10,11]. Vaccine rollout is under way in over 90 countries worldwide with 10 COVID-19 vaccines that have attained emergency-use and/or full marketing authorization, namely Ad5-nCoV, Ad26.COV2.S, AZD1222, BBIBP-CorV, BNT162b2, BBV-152, CoronaVac, mRNA-1237, Sputnik V, and New Crown COVID-19 [12,13].

Notwithstanding the current approvals, there is still limited publicly available data reporting ad interim or final Phase III trial results undergoing appropriate peer-reviewing process. In this respect, most of the results of trials on COVID-19 vaccines have been reported via press releases [14], whereas only four pharmaceutical developers have recently published data from Phase III trials on AZD1222, BNT162b2, mRNA-1237, and Sputnik V [15,16,17,18].

Certainly, in Phase III trials [15,16,17,18] AZD1222, BNT162b2, mRNA-1237, and Sputnik V resulted clinically effective in protecting against COVID-19, by eliciting a large to very large effect in inducing the synthesis of SARS-CoV-2 neutralizing antibodies as reported in a recent network meta-analysis [19]. Nevertheless, some questions are arising concerning the specific differences across these COVID-19 vaccines in their efficacy profile against COVID-19. Therefore, since to date head-to-head comparison trials are neither available nor planned due to the emergency related with the pandemic, we aimed to identify the factors that may potentially influence the clinical efficacy of COVID-19 vaccines for which data from Phase III trials are currently available.

## 2. Materials and Methods

### 2.1. Search Strategy and Study Eligibility

**Figure 1:**
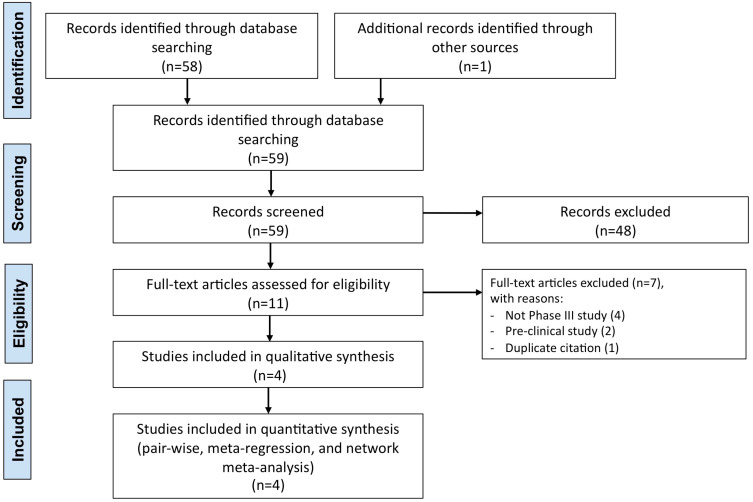
PRISMA-P flow diagram for the identification of the Phase III RCTs included in the quantitative analysis concerning the efficacy of COVID-19 vaccines. COVID-19: Coronavirus Disease 2019; PRISMA-P: Preferred Reporting Items for Systematic Review and Meta-Analysis Protocols; RCT: randomized controlled trial.

The protocol of quantitative synthesis has been registered to the international prospective register of systematic reviews (PROSPERO, registration ID: CRD42021236539), and performed in agreement with the Preferred Reporting Items for Systematic Review and Meta-Analysis Protocols (PRISMA-P) [20]. The relative flow diagram is shown in Figure 1. This study satisfied all the recommended items reported by the PRISMA-P checklist [20].

A comprehensive literature search was performed for Phase III randomized controlled trials (RCTs) written in English and evaluating the efficacy of COVID-19 vaccines at preventing the disease and the potential factors affecting their effectiveness. In this regard, the PICO (Patient problem, Intervention, Comparison, and Outcome) framework was applied to develop the literature search strategy, as previously reported [21]. Namely, “Patient problem” included the prevention of SARS-CoV-2 induced COVID-19; “Intervention” regarded COVID-19 vaccines; the “Comparison” was performed vs. negative control and across COVID-19 vaccines; the assessed “Outcomes” were the efficacy in preventing COVID-19 illness and the evaluation of potential modifying factors influencing the vaccines’ effectiveness.

The search was performed in ClinicalTrials.gov, Cochrane Central Register of Controlled Trials (CENTRAL), Embase, EU Clinical Trials Register, MEDLINE, Scopus, and Web of Science, in order to provide for relevant studies, published up to February 8, 2021. The research string was as follows: (BBIBP-CorV OR (New Crown COVID-19) OR (SARS-COV-2 inactivated vaccine) OR (CoronaVac OR (adsorbed COVID-19 inactivated vaccine)) OR (AZD1222 OR (ChAdOx1 nCoV-19) OR COVISHIELD) OR Ad5-nCoV OR ((Sputnik V) OR Gam-COVID-Vac OR (rAd26-S+rAd5-S)) OR (Ad26.COV2.S OR JNJ-78436735 OR Ad26COVS1 OR VAC31518) OR NVX-CoV2373 OR mRNA-1273 OR BNT162b2 OR (ZF2001 OR (RBD-dimer vaccine)) OR CVnCoV OR QazCovid-in OR INO-4800 OR AG0301-COVID19 OR ZyCoV-D OR (BBV152 OR covaxin) OR SCB-2019 OR CoVLP) AND efficacy. Other sources selected to provide for relevant studies included the “Draft landscape of COVID-19 candidate vaccines” released by WHO [6] and the online archive and distribution server of preprints MedRxiv (available at: https://www.medrxiv.org, accessed on: 8 February 2021). Citations of previous published reviews were checked to select further pertinent RCTs, if any. Literature search results were uploaded to Eppi-Reviewer 4 (EPPI-Centre Software, London, UK), a web-based software program for managing and analyzing data in literature reviews that facilitates collaboration among reviewers during the study selection process.

### 2.2. Study Selection

Phase III RCTs reporting data on the efficacy of COVID-19 vaccines in preventing the illness were selected and included in this quantitative synthesis. The studies in which the immunization schedule, dosing, and route of administration were consistent with those approved were selected and included in the quantitative synthesis.

Two reviewers independently examined the studies, and any difference in opinion concerning the selection of relevant clinical trials from literature searches and databases was resolved by consensus in agreement with a third reviewer.

### 2.3. Data Extraction

Data from the Phase III RCTs included in this quantitative synthesis were extracted from published papers and/or supplementary files. Data were checked for study characteristics and duration, pharmaceutical company, type of COVID-19 vaccine with immunization schedule, dosing and route of administration, number, age, gender of study participants, characteristics, gender and sex of vaccine recipients, rate of COVID-19 cases, items to assess the Cochrane Risk of Bias 2 (RoB 2), and Jadad Score [22].

Data were extracted in agreement with Data Extraction for Complex Meta-anALysis (DECiMAL) recommendations [23]. The inter- and intra-rater reliability for data abstraction was assessed via the Cohen’s Kappa score, as previously described [24]. Briefly, Cohen’s Kappa ≥0.80 indicated excellent agreement, coefficients between 0.61 and 0.80 represented substantial agreement, coefficients between 0.41 and 0.61 moderate agreement and <0.41 fair to poor agreement.

### 2.4. Endpoint

The endpoint of this analysis was to identify the potentially modifying factors influencing the efficacy of COVID-19 vaccines in preventing the illness.

### 2.5. Data Synthesis and Analysis

A pairwise meta-analysis was performed by applying the random-effects model in order to calculate the efficacy of COVID-19 vaccines in terms of protection against the disease compared with negative control. Since data were selected from a series of studies performed by researchers operating independently, and a common effect size cannot be assumed, a binary random-effects model was used in order to balance the study weights and adequately estimate the 95% confidence interval (95%CI) of the mean distribution of vaccination on the investigated variables [25].

Results of the pairwise meta-analysis were expressed as odds ratio (OR) and/or relative risk (RR) with 95%CI. The efficacy of COVID-19 vaccines was reported also as the percentage in prevention of COVID-19 and calculated by using the following formula: “(1 − OR) × 100,” and presented as mean percentage and 95%CI, as previously described [16].

After that, a meta-regression analysis via the random-effect method was carried out to determine the modifying factors that may potentially modulate the efficacy of COVID-19 vaccines. Specifically, only the factors common to all the included studies were selected. The meta-regression was performed by plotting the effect estimates (outcome variables) resulting from the pairwise meta-analysis with the modifying factors (explanatory variables) common to all the included studies [26,27,28,29]. The regression coefficient (slope) obtained from a meta-regression analysis describes how the intervention effect changes with a unit increase in the explanatory variables [28]. This model indicates a positive or negative linear relationship between the intervention effect and the modifying factors only when the regression coefficient is statistically significant.

A network meta-analysis was performed to indirectly evaluate the efficacy in preventing COVID-19 across vaccines in the overall population of participants. A full Bayesian evidence network was used in the network meta-analysis (chains: 4; initial values scaling: 2.5; tuning iterations: 20,000; simulation iterations: 50,000; tuning interval: 10). The convergence diagnostics for consistency and inconsistency were assessed via the Brooks-Gelman-Rubin method, as previously described [30]. Due to the characteristics of parameters besides the available data, the just proper non-informative distributions specified the prior densities, in agreement with the Bayesian Approaches to Clinical Trials and Health-Care Evaluation [31,32]. Since the distributions were sufficiently vague, the reference treatment, study baseline effects, and heterogeneity variance were unlikely to have a noticeable impact on model results. In this condition, GeMTC software automatically generates and runs the required Bayesian hierarchical model and selects the prior distributions and starting values as well, via heuristically determining a value for the outcome scale parameter (i.e., outcome scale S) [33,34]. The posterior mean deviance of data points in the unrelated mean effects model was plotted against their posterior mean deviance in the consistency model in order to provide information for identifying the loops in the treatment network where evidence was inconsistent [35]. The probability that each intervention arm was the most effective was calculated by counting the proportion of iterations of the chain in which each intervention arm had the best relative effect, with the surface under the cumulative ranking curve analysis (SUCRA) representing the summary of these probabilities [36]. The SUCRA is 1 when a treatment is considered to be the best, and 0 when a treatment is considered to be the worst [37].

### 2.6. Assessment of the COVID-19 Rate in the Investigated Populations

The COVID-19 rate was assessed in the investigated populations by referring to the negative control groups. It was calculated as 14-day case rate per 100,000 inhabitants, in agreement with the 14-day notification rate of newly confirmed COVID-19 cases per 100,000 inhabitants, which is part of the Combined Indicator released by the European Centre for Disease Prevention and Control (ECDC) [38]. Detailed information concerning the rank of notification rate is available at: https://www.ecdc.europa.eu/en/covid-19/situation-updates/weekly-maps-coordinated-restriction-free-movement (accessed on 17 February 2021).

### 2.7. Quality of Studies, Risk Bias, and Evidence Profile

The summary of the risk of bias for each included Phase III RCT was analyzed via the Cochrane RoB 2 [39] and Jadad score [22]. The Jadad score ranging from 1 to 5 (score of 5 being the best score) was used to assess the quality of the papers concerning the likelihood of bias related with randomization, double blinding, withdrawals and dropouts [22]. The quality of studies was ranked as follows: score < 3, low quality; score = 3, medium quality; score > 3 high quality.

The test for heterogeneity (I^2^) was performed to quantify the between-study dissimilarity, as previously reported [26], and sensitivity analysis was carried out to identify the studies that introduced moderate to high levels of heterogeneity (I^2^ > 50%) in the quantitative synthesis [40]. The weighted assessment of the risk of bias was analyzed via the Cochrane RoB 2 [39].

The quality of the evidence was assessed in agreement with the Grading of Recommendations Assessment, Development, and Evaluation (GRADE) system, indicating ++++ for high quality of evidence, +++ for moderate quality of evidence, ++ for low quality of evidence, and + for very low quality of evidence [41]. Three reviewers independently assessed the quality of studies, risk bias, and evidence profile, and any difference in opinion was resolved by consensus.

### 2.8. Software and Statistical Significance

OpenMetaAnalyst was used to perform the pairwise meta-analysis and meta-regression, GeMTC for the network meta-analysis, GraphPad Prism to graph the data, GRADEpro GDT to assess the quality of evidence [41], and the robvis visualization software to perform the RoB 2 tool [42,43]. The statistical significance was assessed for *p* < 0.05.

## 3. Results

### 3.1. Study Characteristics

Data obtained from 89,554 healthy adult volunteers of candidate SARS-CoV-2 vaccines were selected from four Phase III RCTs (Table 1). The investigated COVID-19 vaccines included two adenovirus-vector-based vaccines, namely AZD1222 and Sputnik V [15,16] and two lipid nanoparticle-encapsulated mRNA-based vaccines, namely BNT162b2 and mRNA-1273 [17,18].

According to the ECDC geographic risk assessment for COVID-19 transmission, the rate of confirmed COVID-19 cases detected in each of the included Phase III trials indicates a high-risk status, with 50–150 cases per 100,000 inhabitants every 14 days (Table 1).

The inter-rater reliability for data abstraction was excellent before and after the learning process (Cohen’s Kappa 0.96 and 1.00, respectively). The intra-rater reliability produced a Cohen’s Kappa of 1.00 after the learning process.

### 3.2. Pairwise Meta-Analysis

**Figure 2:**
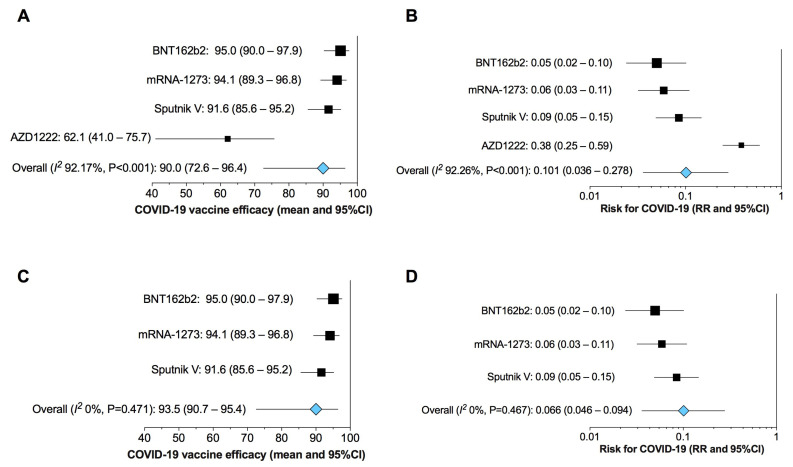
Forest plot of the overall and sensitivity analyses on the efficacy of COVID-19 vaccines in preventing the illness (**A**,**C**) and on the RR for COVID-19 after vaccine administration (**B**,**D**) vs. negative control. COVID-19: Coronavirus Disease 2019; RR: relative risk; 95%CI: 95% Confidence Interval.

The overall pairwise meta-analysis indicated that vaccination against COVID-19 is effective in preventing the disease (overall efficacy: 90.0%, 95%CI 72.6–96.4; *p* < 0.001) (Figure 2A) and reduced the risk of COVID-19 compared to negative control (RR: 0.10, 95%CI 0.04–0.28; *p* < 0.001) (Figure 2B). Both the effect estimates were affected by high and significant heterogeneity (I^2^ overall efficacy 92.17%, *p* < 0.001; I^2^ RR 92.26%, *p* < 0.001) (Figure 2A,B). The sensitivity analysis showed that the study by Voysey et al. on AZD1222 [15] introduced the main source of heterogeneity. As a matter of fact, excluding this study from the analysis permitted to abolish heterogeneity (I^2^ overall efficacy 0%, *p* = 0.047; I^2^ RR 0%, *p* = 0.47) (Figure 2C,D).

### 3.3. Meta-Regression Analysis

The meta-regression analysis was performed by including four potentially modifying factors, common to all the included studies: the type of COVID-19 vaccine, age and sex of recipients, and rate of COVID-19 cases over the observation period of the study in the investigated populations.

There was a significant (*p* = 0.033) linear relationship for the type of vaccine with respect to level of efficacy in preventing COVID-19, but the sensitivity analysis indicated that after excluding the study by Voysey et al. [15], the correlation was no longer significant (*p* = 0.241). Overall, the age and sex of study participants and the rate of new COVID-19 cases did not significantly (*p* > 0.05) influence the efficacy of vaccines in the investigated populations.

**Figure 3:**
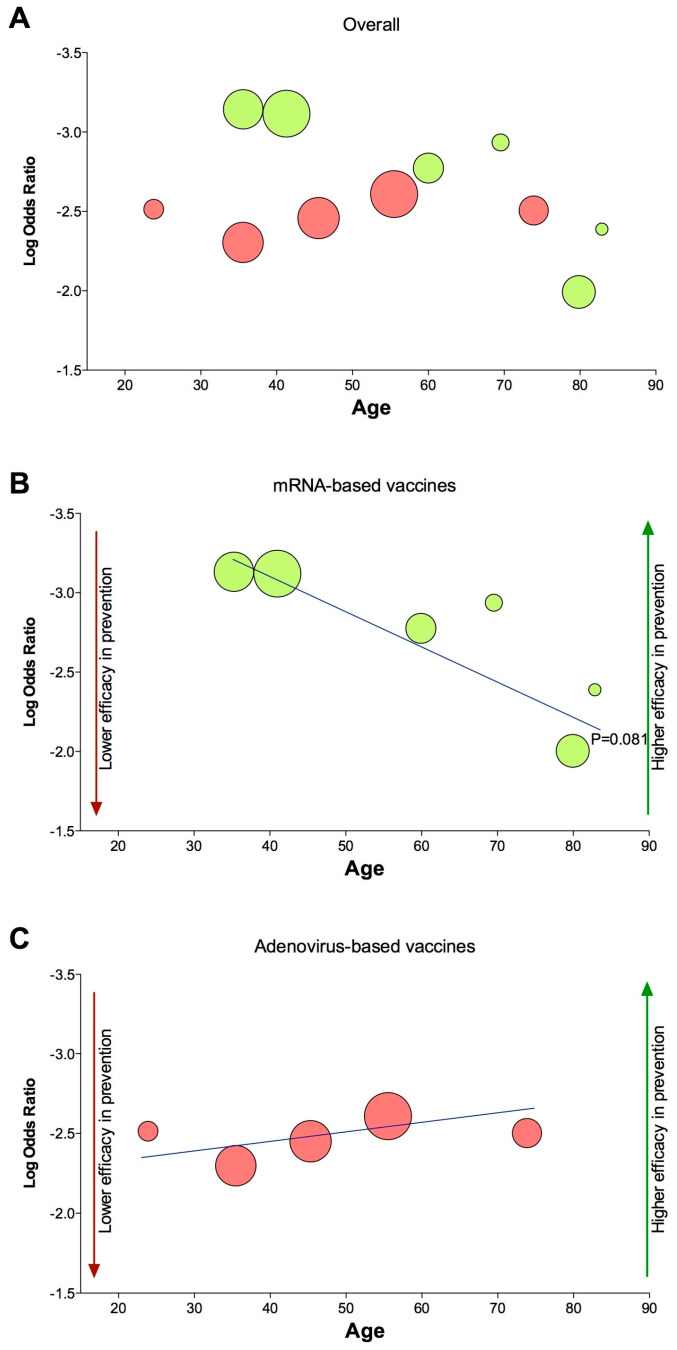
Graphical representation of the meta-regression analysis for age with respect to the changes in the efficacy of COVID-19 vaccines as overall (**A**), mRNA-based vaccines (**B**), and adenovirus-based vaccines (**C**). The size of the circles is proportional to the sample weights. COVID-19: Coronavirus Disease 2019; mRNA: messenger ribonucleic acid.

Although the overall subset analysis according with the type of vaccine reported that age was not a significant (*p* > 0.05) modifying factor (Figure 3A), the results showed a trend toward significance (*p* = 0.081) for mRNA-based vaccines (Figure 3B). Conversely, age was not a significant (*p* > 0.05) modifying factor for adenovirus-based vaccines (Figure 3C). Detailed results of the meta-regression analysis with respect to the type of COVID-19 vaccine, the rate of confirmed COVID-19 cases, and the age and sex of recipients are reported in Table 2.

### 3.4. SUCRA

The ranking resulting from the network meta-analysis identified two distinct clusters of effectiveness. Specifically, the SUCRA provided the following rank of effectiveness: BNT162b2 ≃ mRNA-1273 > Sputnik V >> AZD1222 (Table 3).

### 3.5. Bias and Quality of Evidence

**Figure 4:**
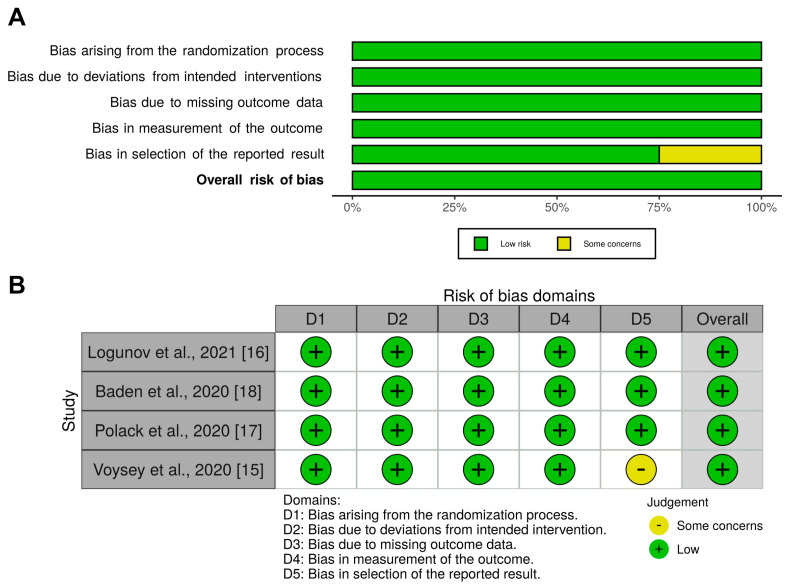
Assessment of the risk of bias via the weighted plot for the assessment of the overall risk of bias (**A**) and the traffic light plot of the risk of bias of each included RCT via the Cochrane RoB 2 tool (**B**) (*n* = 4 studies). The traffic light plot reports five risk of bias domains: D1, bias arising from the randomization process; D2, bias due to deviations from intended intervention; D3, bias due to missing outcome data; D4, bias in measurement of the outcome; D5, bias in selection of the reported result; yellow circle indicates some concerns on the risk of bias and green circle represents low risk of bias. RCT: randomized controlled trial; RoB: risk of bias.

The weighted plot for the assessment of the overall risk of bias by domains is shown in Figure 4A, and the traffic light plot for the assessment of each included study is reported in Figure 4B. All Phase III trials had a low risk of bias for the randomization process, for deviations from intended interventions, missing outcome data, measurement of the outcomes, and selection of the reported results (4 [100.0%]), whereas only one study had some concerns in the domain of selection of the reported results (1 [25.0%]).

All studies (100.0%) were ranked as being of medium- to high-quality in agreement with the Jadad score (Table 1). Three studies were of medium quality (Jadad score = 3) [15,16,17,18] and one was of high quality (Jadad score > 3) [16].

The assessment of the quality of evidence carried out via the GRADE system reported a general high-quality of evidence (++++) for both the overall and sensitivity analyses.

## 4. Discussion

The findings of this analysis demonstrate that although vaccination is effective against COVID-19, the currently approved vaccines for which data from Phase III trials are currently available seem to be characterized by a specific efficacy profile in reducing the risk of symptomatic disease. Furthermore, the overall results of this study indicate that the type of vaccine, age and sex of recipients, and the rate of COVID-19 in the study populations were not modifying factors that may potentially modulate the efficacy of COVID-19 vaccines. Nevertheless, the subset meta-regression analysis performed according with the type of vaccines suggests that, in participants receiving mRNA-based vaccines, the age of recipients could have an influence on the efficacy of vaccines. Conversely, age was not a modifying factor for adenovirus-based vaccines.

Indeed, the trend towards significance for age as being a modifying factor in mRNA-based vaccines should be interpreted with caution. In fact, reporting the efficacy as the logarithm of OR according with meta-regression procedures [44], our analysis indicated that while the level of efficacy was similar for both mRNA-based and adenovirus-based vaccines in older recipients, the protective effect of mRNA-based vaccines against COVID-19 was around a half logarithm greater than adenovirus-based vaccines in younger subjects.

Although a trend towards significance is still an indication of a failure to reach the specific threshold of *p* value set as *p* < 0.05, it is still necessary to step back and evaluate whether what has been detected is actually clinically relevant [45]. The *p* value is perhaps the statistical concept mostly associated with fallacies and misuses in clinical research. Indeed, a common misconception is that in order for a study result to be important, the outcome simply needs to be statistically significant [46]. Estimated effect measures require careful interpretation and precision rather than mechanical significance testing which could lead to intrinsically biased conclusions [45]. As a matter of fact, a non-statistically significant result does not necessarily mean that there is no difference, it rather indicates that the study failed to show a difference, perhaps due to an inadequate sample size. Therefore, it could be speculated that the addition of extra data is likely to result in a significant linear relationship between age and mRNA-based vaccines.

A recent exploratory and ecological study investigated for the first time the association between the composition of age and gender among the whole population and the epidemic features of COVID-19 worldwide [47]. The analysis detected that age was positively correlated to the incidence rate, case fatality rate, and mortality rate of COVID-19 [47]. People over 65 years of age were more prone to develop the disease, whereas a lower incidence rate of COVID-19 was detected in younger individuals [47]. Therefore, a relationship between age and the efficacy of COVID-19 vaccines cannot be excluded and should be further investigated.

The study by Voysey et al. [15] was found to introduce a significant level of heterogeneity in the pairwise meta-analysis. In fact, the efficacy of AZD1222 was considerably lower than in the other trials [16,17,18], a finding confirmed by the sensitivity analysis. Moreover, the SUCRA identified two distinct clusters of effectiveness, with mRNA-based vaccines generally reporting greater ranking score than adenovirus-based vaccines.

Interestingly, a recent pooled analysis of four RCTs performed to assess the timing of the booster dose on efficacy AZD1222 [48] supported the different level of efficacy of this vaccine when compared with the other vaccines included in this quantitative synthesis. Specifically, it resulted that the efficacy of AZD1222 was 82.4% following two full doses with a prime-boost interval of at least 12 weeks [48], whereas the efficacy of BNT162b2, mRNA-1273, and Sputnik V reached values between 91.6% and 95.0% [16,17,18]. This analysis on AZD1222 [48] was not included in the present quantitative synthesis as it provided pooled data from both Phase I/II and Phase III RCTs; thus, it did not comply with the strict inclusion criteria of our study protocol.

The meaningfulness of the reported efficacy results from Phase III trials on BNT162b2 and mRNA-1273 has been extensively and unilaterally questioned [49]. The raised concerns regarded primarily the fact that the studies based their assessment of efficacy profile on the number of confirmed COVID-19 cases, while neglecting symptomatic subjects negative for SARS-CoV-2 [49]. Moreover, those participants who became SARS-CoV-2 positive prior to 7 days after the second dose were excluded from the efficacy analysis for “major protocol deviations,” without giving a specific explanation for this exclusion [49]. As expected, data from recent large real-world evidence [50,51] have rebutted the concerns on the efficacy profile of BNT162b2 and mRNA-1273, by suggesting that vaccination against SARS-CoV-2 not only prevents the clinical signs of COVID-19, but may also limit the transmission of the virus. In other words, a robust immunization campaign can have a powerful impact against the pandemic progression [52,53].

In this regard, a retrospective analysis was conducted on data originating from the Israeli Ministry of Health between March 2020 and February 2021 [50]. The results indicated that almost 6 weeks from the start of vaccination rollout, the number of new COVID-19 infections has dropped by 49% among recipients aged 60 years and older, as compared to 3 weeks earlier; furthermore, the hospitalizations declined by 36% and the number of critically ill people by 29% [50]. Moreover, a recent case control study conducted by the Clalit Institute for Research reported a reduction by 94% in symptomatic COVID-19 cases among 596,618 people who received two doses of the BNT162b2 vaccine, regardless of recipients’ age, compared to matched unvaccinated controls [51].

After health-care workers, COVID-19 vaccination remains a priority in the elderly as they are at much greater risk of death from the disease compared to younger subjects [54]. Nevertheless, there was evidence that in school settings, children and adolescents could become important drivers of SARS-CoV-2 transmission in the general population, especially because of relaxation of mask wearing during extra-curricular activities or with the increase of summertime temperatures [55,56]. The findings from the most comprehensive pediatric study on COVID-19 patients found that children of all ages could carry high SARS-CoV-2 viral loads, especially in the first two days of infection, yet be mildly sick or even asymptomatic [57]. As a consequence, vaccinating at school-age might play a pivotal role to control viral transmission; however, there is still a limited number of studies establishing the epidemiology of COVID-19 and the efficacy of vaccines among children [54].

The main limitations to this analysis are related with the intrinsic weaknesses of the included studies, characterized by differences in the study design and in the reporting of the results. As a matter of fact, the study by Voysey et al. [15] on AZD1222 was not included in the meta-regression analysis for age and sex of recipients, as it did not report vaccine efficacy results across subgroups stratified by demographic characteristics. Moreover, the number of studies included in this analysis was limited due to the strict inclusion and exclusion criteria reported in the protocol and registered “a priori” in PROSPERO, according to the current PRISMA-P guidelines [20]. Indeed, the scientific knowledge concerning the clinical impact of COVID-19 vaccines mostly derives from Phase III clinical trials. However, despite the strong need of clinical knowledge on COVID-19 vaccines and the recent approval for emergency use of several vaccines [19], paradoxically, results from Phase III studies are available from only four published papers [15,16,17,18]. Certainly, this is not a limitation of this research, but it is an intrinsic limitation of the current scientific literature on Phase III studies supporting the clinical efficacy of COVID-19 vaccines. Finally, but not less important, no Phase III studies report data concerning the impact of SARS-CoV-2 variants on the efficacy of COVID-19 vaccines: this is probably a pivotal modifying factor worth to be included as a co-variate in future meta-regression analyses to assess the real clinical impact of vaccines against COVID-19.

Certainly, meta-regression analysis has evolved as a powerful technique for identifying bias from several clinical trials included in a meta-analysis. Thus, meta-regression analysis is usually used to explain whether potential effect modifiers are responsible of the statistical heterogeneity between studies [58]. On the other hand, and as performed in our investigation, meta-regression analysis is an effective tool to assess whether pre-specified co-variates may present themselves as real modifying factors that may modulate efficacy outcomes [59].

## 5. Conclusions

In conclusion, vaccination is effective at helping to control the pandemic, by reducing viral spread and symptomatic transmission, regardless of the type of vaccine, the age and sex, and the rate of COVID-19 of the recipients’ population. Certainly, more work is needed to identify additional modifying factors and address their role in modulating the efficacy against COVID-19, as soon as clinical results on the efficacy profile will be available from Phase III trials for further licensed COVID-19 vaccines.

## Figures and Tables

**Table 1:** Study characteristics of the Phase III RCTs included in this quantitative synthesis.

Study and Year with Reference	Logunov et al., 2021 [16]	Baden et al., 2020 [18]	Polack et al., 2020 [17]	Voysey et al., 2020 [15]
**Trial number Identifier**	NCT04530396	NCT04470427	NCT04368728	ISRCTN89951424; NCT04324606, NCT04400838, NCT04444674
**Vaccine developer**	Gamaleya Research Institute	Moderna/National Institute of Allergy and Infectious Diseases’s Vaccine Research Center	BioNTech/Fosun Pharma/Pfizer	University of Oxford/AstraZeneca
**COVID-19 vaccine (dose and route of administration)**	Sputnik V or Gam-COVID-Vac (1 × 10^11^ viral particles IM)	mRNA-1273 (100 μg IM)	BNT162b2 (30 µg IM)	AZD1222 or ChAdOx1 nCoV-19 (5 × 10^10^ viral particles IM)
**Study characteristics**	Phase III, multicenter, randomized, double-blind, negative-controlled, parallel group	Phase III, multicenter, randomized, single-blind, stratified, parallel group	Phase III, multicenter, randomized, single-blind, negative-controlled, parallel group	Phase III, multicenter, randomized, single-blind, negative-controlled, parallel group
**Study duration with follow-up (weeks)**	~11	~17	~16	~21
**Type of candidate vaccine**	Recombinant adenovirus type 26 vector plus recombinant adenovirus type 5 vector carrying the gene for SARS-CoV-2 full-length spike glycoprotein	LNP-encapsulated nucleoside-modified mRNA vaccine encoding SARS-CoV-2 prefusion-stabilized full-length spike glycoprotein trimer	Three LNP-encapsulated nucleoside-modified mRNA vaccine encoding trimerized SARS-CoV-2 RBD antigen of spike glycoprotein	Replication-defective chimpanzee adenovirus-vectored vaccine expressing full-length SARS-CoV-2 spike glycoprotein gene
**Number of scheduled doses (timing of inoculations)**	Prime and boost inoculation (0, 21 days)	Prime and boost inoculation (0, 28 days)	Prime and boost inoculation (1, 22 days)	Prime and boost inoculation (0, 28–90 days)
**Number of participants**	15,366	28,207	37,086	8895
**Vaccine recipients characteristics**	Healthy adults with negative PCR and IgG and IgM to SARS-CoV-2, with no history of COVID-19 or contact with COVID-19 patients in the preceding 2 weeks before enrolment	Healthy adults or adults with pre-existing stable medical conditions, with no history of SARS-CoV-2 infection	Healthy adults or adults with pre-existing stable medical conditions, with no history of COVID-19, and not taking medications intended to prevent COVID-19	Healthy adults at high risk of exposure to SARS-CoV-2
**Age (mean and range)**	45.3 (18.0–87.0)	51.4 (18.0–95.0)	52.8 (16.0–91.0)	≥18.0
**Male (%)**	61.3	52.7	50.6	41.1
**Rate of COVID-19 cases (number of cases/100,000 inhabitants/14 days)**	343	190	320	279
**Jadad score**	5	3	3	3

COVID-19: Coronavirus Disease 2019; IgG: immunoglobulin G; IgM: immunoglobulin M; IM: intramuscular; LNP: lipid nanoparticle; mRNA: messenger ribonucleic acid; PCR: polymerase chain reaction; RBD: receptor-binding domain; RCT: randomized controlled trial; SARS-CoV-2: severe acute respiratory syndrome-coronavirus-2.

**Table 2:** Model results of the meta-regression analysis performed with respect to the type of COVID-19 vaccine, the rate of confirmed COVID-19 cases, and the age and sex of recipients.

Co-variate	Regression Coefficient, Mean and 95%CI	*p* Value	Modifying Factor
**Vaccine type**			
**Overall**	−1.227 (−2.355–−0.099)	0.033	Yes
**Sensitivity analysis by excluding AZD1222**	−0.430 (−1.149–0.289)	0.241	No
**Rate of COVID-19 cases**	0.001 (−0.013–0.015)	0.933	No
**Age**			
**Overall**	0.014 (−0.008–0.036)	0.215	No
**mRNA-based vaccines**	0.023 (−0.003–0.049)	0.081	No, but detected a trend toward significance
**Adenovirus-based vaccines**	−0.005 (−0.046–0.036)	0.807	No
**Sex**	−0.539 (−1.261–0.183)	0.144	No

COVID-19: Coronavirus Disease 2019; mRNA: messenger ribonucleic acid; 95%CI: 95% confidence interval.

**Table 3:** SUCRA values for the efficacy of COVID-19 vaccines in preventing the illness.

COVID-19 Vaccine	SUCRA (%)
BNT162b2	0.75
mRNA-1273	0.71
Sputnik V	0.62
AZD1222	0.33

COVID-19: coronavirus 2019; SUCRA: surface under the cumulative ranking curve.

## Data Availability

The data presented in this study are available in the article.
